# A protocol for a trial of homeopathic treatment for irritable bowel syndrome

**DOI:** 10.1186/1472-6882-12-212

**Published:** 2012-11-06

**Authors:** Emily J Peckham, Clare Relton, Jackie Raw, Clare Walters, Kate Thomas, Christine Smith

**Affiliations:** 1 School of Healthcare, University of Leeds, Leeds, UK; 2 School of Health and Related Research, University of Sheffield, Sheffield, UK; 3 Barnsley Hospital NHS Foundation Trust, Gawber Rd, Barnsley, UK

**Keywords:** Irritable bowel syndrome, Homeopathic treatment, Attention control, Randomised controlled trial

## Abstract

**Background:**

Irritable bowel syndrome is a chronic condition with no known cure. Many sufferers seek complementary and alternative medicine including homeopathic treatment. However there is much controversy as to the effectiveness of homeopathic treatment. This three-armed study seeks to explore the effectiveness of individualised homeopathic treatment plus usual care compared to both an attention control plus usual care and usual care alone, for patients with irritable bowel syndrome.

**Methods/design:**

This is a three-armed pragmatic randomised controlled trial using the cohort multiple randomised trial methodology. Patients are recruited to an irritable bowel syndrome cohort from primary and secondary care using GP databases and consultants lists respectively. From this cohort patients are randomly selected to be offered, 5 sessions of homeopathic treatment plus usual care, 5 sessions of supportive listening plus usual care or usual care alone. The primary clinical outcome is the Irritable Bowel Syndrome Symptom Severity at 26 weeks.

From a power calculation, it is estimated that 33 people will be needed for the homeopathic treatment arm and 132 for the usual care arm, to detect a minimal clinical difference at 80 percent power and 5 percent significance allowing for loss to follow up. An unequal group size has been used for reasons of cost. Analysis will be by intention to treat and will compare homeopathic treatment with usual care at 26 weeks as the primary analysis, and homeopathic treatment with supportive listening as an additional analysis.

**Discussion:**

This trial has received NHS approval and results are expected in 2013.

**Trial registration:**

Current Controlled Trials ISRCTN90651143

## Background

Irritable Bowel Syndrome (IBS) is a common, chronic disorder that affects approximately 10% - 22% of the population [1]. It is characterised by abdominal pain or discomfort and altered bowel habits, and may also be accompanied by bloating, nausea and vomiting and early satiety. IBS is a functional gastrointestinal disorder. Functional gastrointestinal disorders are difficult to treat because no single aetiology is known and thus treatment is directed at controlling symptoms, using pharmacological and non-pharmacological approaches. There are an estimated 240,000 primary care consultations per year in the UK of new cases of IBS [2] and the economic costs of IBS in primary care are estimated to be over £200 million [3]. IBS is diagnosed using the Rome criteria, the most recent being Rome III [4], or on the basis of clinical symptoms with the absence of alarm signs (signs that indicate the potential presence of a serious disease). There are four subtypes: IBS-C constipation predominant, IBS-D diarrhoea predominant, IBS-M mixed and IBS-unspecified.

The prognosis for recovery with IBS is poor. A one year prospective evaluation found that although 50% of patients improved over the year, improvement was minor in terms of IBS symptoms such as pain, constipation and diarrhoea [5]. This could lead to the patients’ quality of life being adversely affected resulting in depressed mood, sleep disturbance and fatigue [6,7]. IBS is associated with high healthcare utilisation costs and loss of productivity [8,9]. Despite much research into both psychological and pharmacological treatments there is no consensus as to its optimal treatment [10].

A significant proportion of patients with gastrointestinal disorders use complementary or alternative medicine, between 11% and 43% according to one systematic review [11]. Gastroenterology problems are the fourth most common referral to NHS homeopathic hospitals [12] and one of the eight most common conditions treated by NHS homeopaths in general practice [13], with irritable bowel syndrome being the tenth most common condition seen by NHS homeopathic hospitals [14]. There is currently a degree of scepticism regarding homeopathic treatment with claims that it is a placebo treatment and therefore unethical [15]. Much of the criticism focuses on the opinion that the homeopathic medicine is no more than a placebo [16] and it is the long consultation time with an empathetic practitioner that leads to any perceived effectiveness of homeopathic treatment [17].

A literature search for trials of homeopathic treatment for IBS using the search terms ‘irritable bowel syndrome’, or ‘irritable colon’ and ‘homeopathy’ identified many case reports e.g. a clinical audit [18], one consecutive case series [19] and three randomised controlled trials [20-22]. Two randomised controlled trials (RCTs) assessing the effectiveness of one specific homeopathic medicine (asafoetida) reported positive results associated with homeopathic medicine compared to placebo [20,21] and one found no difference between homeopathic treatment and usual care [22]. In the consecutive case series [19], twenty out of twenty five patients reported an improvement in the intensity and frequency of their symptoms. In spite of its popularity in the treatment of gastroenterology disorders there is a lack of robust evidence as to the effectiveness of homeopathic treatment for IBS and thus there is a need for further research into the clinical effectiveness of homeopathic treatment for patients with IBS.

**Figure 1:**
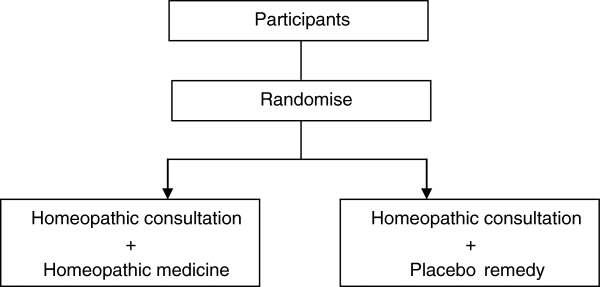
Standard RCT for homeopathic treatment.

The majority of previous trials of homeopathic treatment focus on the homeopathic medicine as the key ingredient and as such compare changes in health of subjects having a homeopathic consultation plus a homeopathic medicine with changes in health in subjects having a homeopathic consultation plus a placebo medicine, Figure 1 shows a schematic of the standard RCT for homeopathic treatment. Studies using the design depicted in Figure 1 are only able to assess the effect of the homeopathic medicine and are not able to assess the effect of homeopathic treatment as a package, (the homeopathic consultation plus the homeopathic medicine). There has been much debate as to whether or not this is an appropriate design to determine the efficacy of homeopathic treatment [23], in part because the traditional homeopathic medicine versus placebo medicine design fails to take into account any aspects specific to the homeopathic consultation. Furthermore the homeopathic approach is best understood as a complex intervention with component parts (consultation and medicine) consequently the design of an appropriate control is less straightforward than would be the case if a drug therapy alone were being evaluated. Pragmatic trials comparing homeopathic treatment (as a package of consultation plus homeopathic medicine) to usual care provide a means of assessing the effect of homeopathic treatment as a package. However the lack of a comparison intervention in the homeopathic treatment plus usual care versus usual care design leads to the question as to whether any observed effectiveness of homeopathic treatment is due to non-specific effects of spending time with an empathetic practitioner. A possible solution to this problem is to compare homeopathic treatment to an “attention control” designed to control for the time and attention that the patient spends with/receives from the homeopath. Whilst other studies have been conducted to assess the effect of the homeopathic consultation, [17,24] as yet no studies have attempted to compare the whole package of homeopathic treatment to a control intervention as a means of assessing the effects of homeopathic treatment.

For this study supportive listening was chosen as the “attention control”. Supportive listening is a non-specific treatment that has been used in previous trials of psychological therapies for IBS [25]. It has in the past been used to control for the non-specific aspects of therapy including the time that the patient spends with the practitioner, empathy and positive regard [26]. Supportive listening is not formal counselling *per se*[27], and although based on the theories of Carl Rogers and utilising the skills of active listening, it does not include the more advanced Rogerian skills such as challenging, problem clarification and accurate understanding [27]. Including supportive listening as one of the arms in a RCT of homeopathic treatment allows homeopathic treatment to be compared to spending time with a caring and empathetic practitioner. Supportive listening was chosen as an “attention control” because it contains the non-specific factors of homeopathic treatment such as the opportunity for disclosure and empathy without containing the specific effects such as the homeopathic medicine and the in-depth enquiry into bodily complaints. A supportive listening arm will be included in this trial to test the feasibility of supportive listening as an attention control for homeopathic treatment, in the treatment of IBS. In addition, a usual care arm is included in the trial, this is to put the results into context and allow an assessment to be made as to whether homeopathic treatment in addition to usual care offers any benefits over usual care alone.

Therefore, in this study we are attempting to assess whether homeopathic treatment (consultation plus homeopathic medicine) is more effective than usual care alone and whether homeopathic treatment is any more effective than spending time with an empathetic and caring practitioner as assessed by change in IBS symptom severity score (IBS-SSS) between baseline and 26 weeks. This study aims to test the whole intervention of “homeopathic treatment”. It is not aiming to, and nor is it designed to, give new insights into the question as to the effects of homeopathic medicines *per se*. Results of this study will report the clinical effectiveness of homeopathic treatment plus usual care compared to usual care alone, and provide information on the feasibility of including a supportive listening arm as an attention control for homeopathic treatment, for patients with IBS. Additionally the supportive listening arm will provide information on effect size and variation, information which will enable future investigators to more accurately estimate the sample size required for a full scale trial comparing homeopathic treatment to supportive listening.

The aims of this study are:

▪ to evaluate the clinical effectiveness of homeopathic treatment plus usual care as compared to usual care alone for patients with IBS.

▪ to test whether supportive listening is a feasible attention control for homeopathic treatment.

## Methods

This study uses the Cohort multiple RCT design [28]. This design was chosen because it allows a number of RCTs to be carried out using a single cohort of people with IBS, see below. This permits increased comparability between trials conducted within the cohort and allows for data to be collected on the natural history of the condition.

### Identification and recruitment

**Figure 2:**
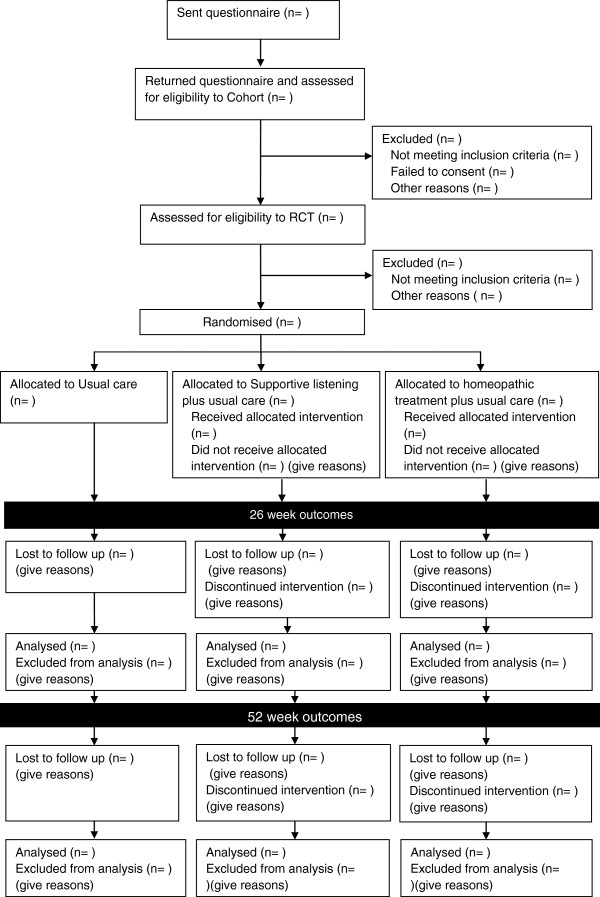
Flow diagram for trial showing intervention and non-intervention arms.

The Cohort multiple RCT design involves the recruitment of a cohort of people to an observational study. These people agree to be observed over time for research purposes through completing questionnaires. Those in the cohort may then subsequently be identified as eligible to take part in one or more RCTs. The cohort used in this study (termed the Barnsley irritable bowel syndrome cohort (BIBSC)) was set up specifically for this study. However there is the potential for other RCTs to be carried out in the future using the BIBSC. Once identified as eligible to take part in this RCT participants are either randomly selected to be offered a treatment (in this instance supportive listening or homeopathic treatment) or randomly selected to make up the control arm of the RCT. Patients who are randomly selected to one of the two treatment arms give their consent to treatment at this point. Figure 2 shows a flow diagram of this process.

Potentially eligible participants for recruitment to the IBS cohort are identified through two routes:

Primary care: GP databases are searched for the names and addresses of patients aged over 18 with a diagnosis of IBS or given medications used to treat IBS symptoms and who have consulted their GP for IBS within the last two years.

Secondary care: Patients are identified by Gastroenterological clinicians at Barnsley Hospital.

The Episode study [29] has shown that the burden of symptoms of patients with IBS in primary care is similar to the burden of symptoms of those in secondary care; therefore no attempt will be made to stratify by recruitment route.

Those identified as potentially eligible for recruitment to the IBS cohort are sent a letter inviting them to take part in an observational study along with a questionnaire to complete and return. This questionnaire forms the baseline questionnaire and is used to identify those eligible for the RCT and the cohort.

**Figure 3:**
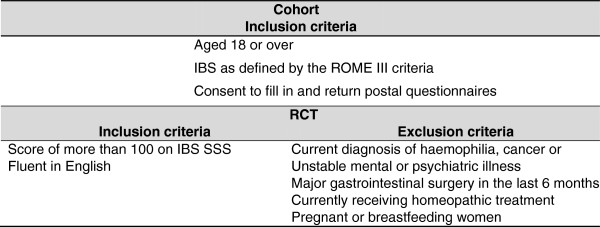
Inclusion and exclusion criteria.

There are two sets of inclusion criteria for this study, inclusion criteria for the cohort and inclusion criteria for the RCT study. Figure 3 gives the inclusion and exclusion criteria for both the RCT and the Cohort.

### Randomisation and blinding

Eligible participants are randomised to, homeopathic treatment plus usual care or supportive listening plus usual care or usual care alone by the shuffling of sealed opaque envelopes containing the allocation. Questionnaires from participants consenting and meeting the eligibility criteria are taken one at a time, at the same time a sealed opaque envelope containing the allocation is taken from the top of the shuffled pack and opened and the allocation noted. This is carried out by an independent administrator at the University of Sheffield, in the presence of another independent administrator. Letters offering treatment are then sent to those randomly allocated to one of the two active treatment arms.

Neither the nature of the interventions in this study nor the study design allows for the masking of the therapists or the participants. However data are inputted and analysed blind to treatment allocation.

### Interventions

Participants allocated to the offer of homeopathic treatment or the offer of supportive listening are offered up to five one hour appointments at Barnsley Hospital over a six month period. Due to the pragmatic nature of this trial it will be for the participants to decide as to whether or not they attend all five sessions, however they will be encouraged to attend appointments as would be the case in usual practice. All participants remain in the care of their GP and continue to receive their usual NHS treatments. Homeopathic treatment involves a homeopathic consultation followed by the prescription of a homeopathic medicine and is provided by professional homeopaths registered with the Society of Homeopaths with at least five years experience. The homeopaths providing treatment in this study do not have a particular specialism in IBS, however they have taken part in a previous trial assessing the effectiveness of homeopathic treatment for fibromyalgia [30]. The homeopaths can choose from any of the homeopathic medicines in the homeopathic pharmacopeia.

Supportive listening is based on the theories and counselling techniques of Carl Rogers [31] and involves active listening skills such as empathising, reflecting, summarising and paraphrasing. In the sessions patients are able to talk about their physical symptoms as well as any emotional issues and possible ways of coping with these better. It provides patients with the opportunity to express themselves and feel heard in a non-judgemental environment. The sessions are delivered by trained psychotherapists registered with either the British Association for Counselling & Psychotherapy or the United Kingdom Register of Counsellors and Psychotherapists. For intervention fidelity, and to ensure that the practitioners delivering the supportive listening are delivering supportive listening rather than Cognitive Behavioural Therapy or any other counselling intervention, a random selection of sessions is taped and assessed by an independent assessor, who will describe the approaches used.

The patients’ perceptions of the effects of interventions and their acceptability to patients will be assessed in a nested qualitative study exploring patient and practitioner experiences of delivering and receiving treatment within the trial. This qualitative study will take the form of hour-long semi-structured interviews aimed at eliciting information about what, it anything patients perceive to have led to any improvement in their IBS or general health. The aim being to explore patients perceptions of the treatment they received, what, if anything they believe led to any effectiveness the treatment and their views on the acceptability of the treatment. It is the intention to publish full details of the qualitative study at a later date.

### Outcome measures

The primary outcome measure is the IBS Symptom Severity Scoring (IBS-SSS) [32]. Secondary outcome measures are the Hospital Anxiety and Depression scale (HADs) [33] and EQ-5D [34]. The EQ-5D was chosen as the quality of life measure rather than a disease specific measure such as the IBS quality of life measure (IBS-QOL) [35] because the EQ-5D was required for the cost effectiveness component of this study. Therefore to reduce the burden of filling out multiple questionnaires on patients it was decided to utilise solely the EQ-5D rather than an additional disease specific health related quality of life measure.

Cost effectiveness is calculated using the EQ-5D, a measure of health related quality of life along with data collected on medication and health services usage and absences from work.

The credibility of the treatments to the patients is assessed using a validated measure originally designed by Borkovec [36] and modified by Drossman [37] for IBS. A single measurement at 26 weeks is made using the CARE empathy measure for participants in either the homeopathic treatment or supportive listening arms [38]. This is a measure of the practitioner’s empathy as perceived by the patients.

Outcomes are sought by postal questionnaire at baseline, 26 weeks and 52 weeks. The primary endpoint of this study is 26 weeks, whilst the 52 week questionnaire will provide data on the longer term effectiveness of the interventions.

### Sample size

The RCT sample size calculation has been based on the primary outcome, which is the change in IBS-SSS between baseline and 26 weeks. The IBS-SSS is based on a series of visual analogue scales (VAS) and has been validated for use in assessing IBS severity. It is scored between 0 and 500 and a higher score indicates more severe IBS. A change of 50 points is considered to be a clinically relevant change [32]. It has been suggested that VAS scales are of greater value when used to determine change within individuals rather than being used to compare scores at a set time frame across a group of individuals [39]. Therefore change in IBS-SSS is used rather than an endpoint score. This study has been powered to detect a difference between homeopathic treatment and usual care. Powering to detect a difference between homeopathic treatment and supportive listening was considered but found to be beyond the resources of this study. However the study will still be able to provide information on the feasibility of supportive listening as an attention control for homeopathic treatment.

A 4:1:1 ratio of usual care: homeopathic treatment: supportive listening was chosen for this trial because of cost limitations. The cost of providing homeopathic treatment or supportive listening was higher than costs associated with the usual care arm of the trial. Using unequal group size provides a means of reducing the cost of a trial whilst maintaining power [40]. A power calculation was carried out using sample size determination software called PS Power [41]. Assuming use of an independent *t* test to compare groups, power 80%, significance level 5%, clinically relevant change of 50 on the IBS-SSS [32] and based on previous RCTs [42-44] standard deviation of 85, ratio of usual care to homeopathic treatment of 4:1, 29 participants are required for the homeopathic treatment and supportive listening arms and 116 for the usual care arm for this comparison. In a previous IBS study [45] it was found that there was a 13% loss to follow up and taking this into account increases the estimated sample size to 33 for the homeopathic treatment and supportive listening arms and 132 for the usual care arm. Therefore in total 198 people will be required for this study, 33 people for each of the homeopathic treatment and supportive listening arms and 132 people for the usual care arm.

### Data analysis

Descriptive statistics and a CONSORT type flow diagram [46] are used to describe the flow of all participants through the trial. The following baseline patient characteristics are reported for all participants meeting the RCT criteria: age, IBS-SSS, HAD score, number of prescribed/self prescribed medications, medication total and EQ-5D score. Baseline data on those who accepted the offer of treatment and those who refused are compared to assess whether there are any differences between those who accepted and those who declined treatment.

Data are analysed on an intention to treat basis using a 2-sided 5% significance level. All participants are included in the group they were randomised to regardless of whether they received their allocated treatment. The primary outcome is the mean change in IBS-SSS between baseline and 26 weeks assessed using analysis of covariance adjusting for baseline scores. The percentage of those achieving a clinically relevant change of 50 points on the IBS-SSS will be reported for each of the three groups. The mean change in HADS between baseline and 26 weeks will also be assessed using analysis of covariance adjusting for baseline scores. All data are tested for normality and where normality is not met the equivalent nonparametric tests will be used.

## Discussion

This research received ethics approval from the Leeds East Research Ethics Committee (10H/1306/73).Results are expected in 2013.

## Competing interests

Clare Walters and Jackie Raw work part time as self-employed homeopaths.

## Authors’ contributions

EP has taken the lead role in drafting and revising the manuscript. EP and CR wrote the protocol. JR, KT, CS and CW have all played significant roles in developing the study protocol and have helped revise the manuscript critically. All authors read and approved the final manuscript.

## Pre-publication history

The pre-publication history for this paper can be accessed here:

http://www.biomedcentral.com/1472-6882/12/212/prepub
